# The Metabolic and Analytical Changes of Healthy Volunteers upon Intake of Portuguese Extra Virgin Olive Oil: A Comparison Study between Pre- and Post-Intervention

**DOI:** 10.3390/nu15153351

**Published:** 2023-07-28

**Authors:** Marta Correia, Inês Moreira, Jane El Maghariki, Tânia Manuel, Paulo Alves, Rui Barros, Ana Gomes

**Affiliations:** 1 CBQF—Centro de Biotecnologia e Química Fina—Laboratório Associado, Escola Superior de Biotecnologia, Universidade Católica Portuguesa, Rua Diogo Botelho 1327, 4169-005 Porto, Portugal; inesdcmoreira@gmail.com (I.M.); janemaghariky@gmail.com (J.E.M.); amgomes@ucp.pt (A.G.); 2 Centro de Investigação Interdisciplinar em Saúde—Instituto de Ciências da Saúde, Universidade Católica Portuguesa, 4169-005 Porto, Portugal

**Keywords:** extra virgin olive oil, cardiovascular disease, Mediterranean diet

## Abstract

(1) Background: Extra virgin olive oil (EVOO) is studied mostly for its health benefits in preventing non-communicable chronic diseases, particularly within a Mediterranean dietary pattern. However, few studies have addressed the effect of EVOO in healthy individuals, prior to an established disease. This study aims to evaluate the impact of Northern Portuguese polyphenol-rich EVOO (NPPR-EVOO) consumption on various important clinical parameters in healthy adult volunteers. (2) Methods: This quasi-experimental intervention study assessed the impact of NPPR-EVOO for a period of 100 days. Serum total cholesterol, HbA1c, HDL-c, LDL-c, and CRP, and anthropometric measures—waist and hip perimeters, hand grip strength, and body fat—were assessed and food logs were analyzed. (3) Results: Serum HbA1c (5.12 ± 0.32%; 4.93 ± 0.24, *p* = 0.000) and LDL-c (96.50 ± 28.57 mg/dL; 87.41 ± 31.38 mg/dL, *p* = 0.017) significantly decreased following NPPR-EVOO. Also, daily energy significantly increased, but no changes in other dietary parameters, or anthropometry, were seen. Adherence to the Mediterranean diet did not explain the differences found in individuals regarding serum lipid profile and HbA1c, reinforcing the role of EVOO’s effect. (4) Conclusions: NPPR-EVOO lowered the serum levels of LDL cholesterol and HbA1c, providing clues on the effect of EVOO-putative health benefits. These results pave the way for a deeper exploration of EVOO as a functional food.

## 1. Introduction

Olive oil (OO) is a food item holding significant value for local economies in the Mediterranean region, such as Portugal [1], and stands out as one of the most extensively studied products in terms of its health benefits and contributions to non-communicable chronic diseases [2]. Furthermore, extra virgin olive oil (EVOO), which is obtained exclusively from olives using mechanical or other physical means, retains the oil’s characteristics, excluding the oils obtained using solvents or re-esterification methods, and has interesting nutritional and sensorial characteristics, with a maximum free acidity of oleic acid of no more than 0.8 g per 100 g (0.8%) [3].

Portugal is known for its high-quality EVOO and great variety in olive trees. One of the most significant examples of this is the monovarietal Santulhana EVOO, produced in the northern region of Trás-dos-Montes. In the scope of a previous project (“Bio-n2-value—Biological tools to add and defend value in the main agri-food chains”, NORTE-01-0145-FEDER-000030), it was demonstrated that similar monovarietal EVOOs from different locations in the Trás-os-Montes region in the north of Portugal revealed different lipid and polyphenolic compositions at concentrations that may support EVOO’s biological functionality [4]. These significant differences in EVOO composition may explain why high-polyphenol EVOO ameliorates certain cardiovascular disease (CVD) risk factors such as cholesterol metabolism, or oxidative-stress-related outcomes, as opposed to an EVOO low in such bioactive molecules [5]. This Northern Portuguese polyphenol rich-EVOO (NPPR-EVOO) has distinctive characteristics arising from its unique cultivar and environment conditions, making up the saponifiable fraction with 73% monounsaturated fatty acids (MUFA) (mainly oleic acid), 16.5% saturated fatty acids (mainly palmitic acid), and 10.6% polyunsaturated fatty acids (PUFAs) (mainly linoleic acid), and a minor unsaponifiable fraction composed of 224.9 μg GAE/g total phenolic compounds with (a) oleuropein, the most prominent phenolic compound (more than 90% of the total polyphenols fraction), (b) tyrosol, and (c) hydroxytyrosol. The importance of these molecules on metabolism, modulation of inflammatory responses, and gene expression should be emphasized [6,7,8]. Moreover, a serving size of 20 g of EVOO contains 4.1 mg of hydroxytyrosol and its derivatives, which has a significant antioxidant capacity [9,10].

EVOO’s phenolic profile and lipid make up reflect its authenticity and origin and have been studied in clinical trials by numerous authors for its beneficial health attributes [11,12]. Indeed, the range of EVOO’s health benefits derive from its specific and unique nutritional composition. Importantly, EVOO is also an important hallmark of the Mediterranean diet (MedD), known to have cardio-protection effects, in which its consumption is known to be inversely associated with the risk of stroke and coronary heart disease [13]. Additionally, the phenolic content of EVOO will affect cholesterol lipoproteins differently depending on its composition [5,14], such as serum lipid profiles, decreasing LDL-cholesterol, and increasing HDL-cholesterol [15], as well as modulating age-related HDL-cholesterol levels [16], all of which are important modifiable risk factors for CVD [17]. In addition, the consumption of EVOO was shown to improve postprandial glucose [18], lower the risk of diabetes, ameliorate metabolic and inflammatory biomarkers [19,20], and decrease gestational diabetes in pregnant women [21]. EVOO has also been shown to alter gut microbiota due to its phenolic compounds potentially modifying the oxidative status of the host’s intestinal barrier, inflammation, and immune response [22]. Additional benefits include treating gastrointestinal disorders like ulcerative colitis [23].

Nonetheless, the literature is not consistent, as some studies lack clarity on whether the observed health benefits arise from EVOO’s properties, or because of a dietary model, such as MedD [24,25].

Therefore, this study aims to evaluate the impact of the consumption of polyphenol-rich EVOO, with a high profile of bioactive molecules [7], on various important clinical parameters, such as anthropometric measurements and serum biomarkers that play a role in chronic diseases, particularly CVD, in healthy adult volunteers willing to participate in this study.

## 2. Materials and Methods

### 2.1. Study Design and Recruitment

A quasi-experimental design was conducted to establish a cause-and-effect relationship between an independent (NPPR-EVOO intake) and dependent variable (metabolic and clinical variables). This study did not rely on random assignment mainly due to practical reasons, using a convenience sample of 37 participants recruited mostly from the Universidade Católica Portuguesa, Porto campus. The convenience sample was a quick and convenient method and all 37 participants willing to participate met the inclusion criteria: population of healthy volunteers, aged between 18 and 55 years old, with a body mass index between >18.0 kg/m^2^ and (BMI) ≤ 30 m/kg^2^ and who normally included olive oil in their diet. The study aimed to assess the impact of a ±30 mL daily intake of an NPPR-EVOO for a period of 100 consecutive days, which is also used in similar clinical studies [26].

This study used a quasi-experimental design to establish a cause-and-effect relationship between the intake of NPPR-EVOO on important anthropometric and clinical/analytical analysis. Participants were not randomly recruited and were all assigned to the group of NPPR-EVOO. The NPPR-EVOO was previously analyzed, chemically and biologically, and the results were presented elsewhere [9,10]. Briefly, the NPPR-EVOO was shown to present high levels of polyphenols known for harboring a significant antioxidant effect. The fatty acid profile was rich in oleic acid (>70%), highlighting the important amount of MUFAs. The monovarietal NPPR-EVOO used in this study presented significant levels of polyphenols, close to the EFSA health claim requisites shown to be beneficial in the protection of LDL-C particles from oxidative damage (5 mg of hydroxytyrosol, oleuropein and tyrosol, per 20 g of EVOO). Hence, a daily intake of ±30 mL of NPPR-EVOO contributes to blood lipid oxidation protection through polyphenol activity [10,27].

The university’s social media accounts (LinkedIn, Instagram, and Facebook) were used to promote the study, providing the information and links where potential volunteers could share their contact details to enroll in the study. The investigation team then applied the exclusion criteria and decided the eligibility of volunteers in a face-to-face interview, along with the nutritionist and nurse team.

Applicable exclusion criteria were current active infections, medications (antidepressants, antibiotics, anti-inflammatories, angiotensin-converting enzyme inhibitors, insulin, and oral anti-diabetic medication), dietary supplements (minerals and oligoelements, antioxidants, *n*-3 polyunsaturated fatty acids, *n*-6 polyunsaturated fatty acids, and *n*-9 fatty acids), the presence of chronic/metabolic/autoimmune diseases such as diabetes, arterial hypertension, cancer, inflammatory diseases, pregnancy, hormone replacement therapy, and nutritional supplements like selenium and omega 3, 6, and 9. The study was submitted to and approved by the Ethical Committee Board for Health from the Universidade Católica Portuguesa (Project nº 171-CES/UCP) and was submitted and approved on the clinical trial platform NCT05852275 (https://clinicaltrials.gov/, accessed on 29 June 2023).

### 2.2. Data Collection Tools and Techniques

The study included two different time point assessments: the initial assessment (time point 0, day 0) before the NPPR-EVOO intake, and the final time point (100 days at the end of the study) after NPPR-EVOO consumption (100 days of NPPR-EVOO ± 10 days). Both initial and final assessments were conducted in a face-to-face interview (multidisciplinary team, including nurse and nutritionist). Although not presented here, there were other moments including the face-to-face interview and moments between the health team and participants, important to assess compliance with the 30 mL-NPPR-EVOO intake and for overall health assessment.

Serum biomarkers were assessed using a capillary blood sample collected from a finger using a single-use lancet which was analyzed using the Cobas^®^ b101 device (Roche), a piece of point-of-care equipment that allows for the rapid quantitative measurement of glycated haemoglobin (HbA1c), total cholesterol, high-density lipoprotein cholesterol (HDL), low-density lipoprotein cholesterol (LDL-C), and C-reactive protein (CRP) using specific sensor kits. Serum biomarkers were obtained in the first and final assessments. The anthropometric measurements were obtained according to standardized procedures by a trained team. Briefly, body weight was measured using an electronic weighing scale and height with a stadiometer. According to the World Health Organization (WHO) criteria, a BMI of less than 18.5 kg/m^2^ is considered underweight, 18.5–24.9 kg/m^2^ is normal, 25–29.9 kg/m^2^ is overweight, and 30 kg/m^2^ and above is obese [28]. Body fat percentage (%) and free fat mass (%) were also measured using an Inbody 720 BIA; waist circumference (WC) was measured using a flexible measuring tape as well as hip circumference (HC). Participants kindly removed heavy clothing from the waistline and stood with their backs straight. The tape was aligned at the top of the hip bone (iliac crest) parallel with the edge of the last palpable rib. WC measurement was taken at the end of a normal expiration and approximated to the nearest 0.5 cm. The WC cutoffs were considered high if >94 cm for men and >80 cm for women [29]. Obese and underweight participants, classified in accordance with the definition of WC, were also excluded.

Furthermore, both the PREDIMED [30] and the IPAQ (International Physical Activity Questionnaire—Short Form) [31] questionnaires were administered at the baseline and final assessment stage. Importantly, the study did not aim to change or influence dietary habits or food choices, and participants were encouraged to maintain whatever diet they practiced. Additionally, participants were instructed to maintain a 3-day food diary at both timepoints which was followed by data processing with the Nutrium^®^ nutritional computer program.

### 2.3. Trial Outcomes

The trial was registered in March 2023 with the clinical trials registration platform. Primary outcomes of the trial include changes in total cholesterol, LDL-cholesterol, HbA1c from baseline to follow-up, body weight and anthropometry changes, adherence to MedD throughout the study, and systolic and diastolic blood pressure.

### 2.4. Statistical Analysis

Data on dietary intake was imported to Statistical Package for Social Sciences (SPSS) version 23. Participants were classified as adhering to MedD (PREDIMED score ≥ 10) and non-adhering to MedD (PREDIMED score < 10). The log function was obtained for all skewed distributed variables in order to normalize the data. The parametric *t*-test was used to compare the mean levels of anthropometry, biochemical and metabolic data, and dietary data among the two groups of NPPR-EVOO consumers. Multiple linear regression and logistic regression were used to adjust physical activity, energy intake, age, and sex with BMI and WC. Additionally, to better explore the role of NPPR-EVOO on clinical variables, we conducted a statistical analysis on the individuals that did not change their physical activity, nor their food habits expressed as their PREDIMED score, so that more homogeneity would be achieved regarding the effect of NPPR-EVOO. Habitual intake was estimated for three days using detailed 24-h recall, and the mean level was calculated. Comparisons were made using χ^2^ test, Student’s *t*-test, or non-parametric tests as appropriate. Spearman non-parametric correlations were used to assess relationships. Statistical significance was determined for *p* < 0.05.

## 3. Results

**Figure 1:**
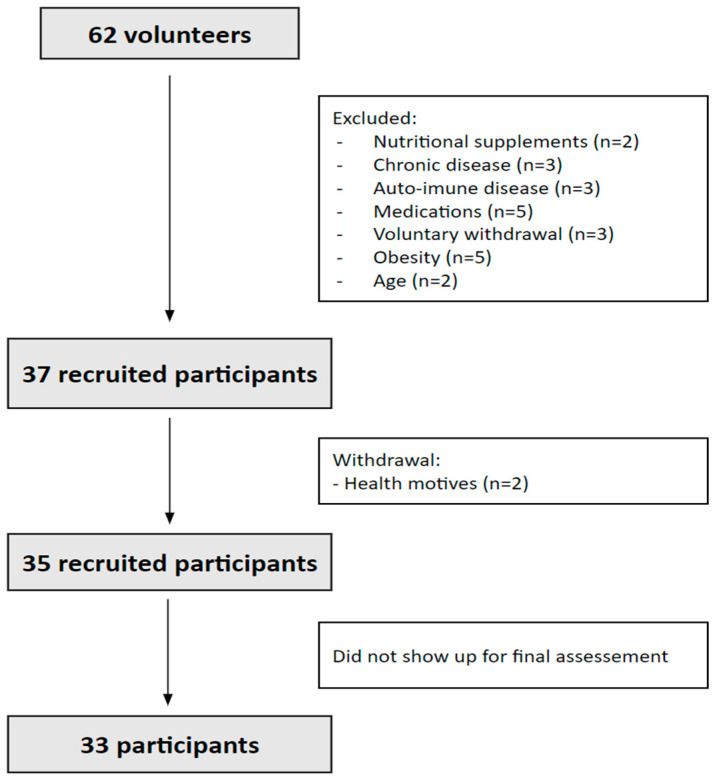
Recruitment of the participants.

In this quasi-clinical study, a total of 37 participants were initially recruited (Figure 1).

As a result, the final sample of the study consisted of 33 individuals (8 men and 25 women), aged between 19 and 55 (average 33.5 ± 11.2 years); all individuals already consumed common OO in their daily meals. Overall, there were no significant changes regarding physical activity when comparing the beginning and the end of the study. However, the inclusion of NPPR-EVOO increased participants’ adherence to the MedD, expressed by a higher PREDIMED score at the end of the study. Also, at the end of the study, individuals within the range of normal-high blood pressure decreased. No significant changes were observed regarding anthropometry and body weight, although there was a tendency toward a lower percentage of total fat mass in women at the end of the study (Table 1). Interestingly, some serum analytical parameters changed at the end of the study, such as HbA1c (5.12 ± 0.32; 4.93 ± 0.24, *p* = 0.000) and LDL-c (92.63 ± 24.53; 83.23 ± 27.63, *p* = 0.024). Nevertheless, daily calories increased significantly (*p* < 0.05) throughout the study, but no significant changes (*p* < 0.05) in the profile of the consumption of fatty acids, lipids, or cholesterol were observed. All the other parameters did not show any significant changes between the beginning and the end of the study.

Because NPPR-EVOO has been associated with cardiovascular risk reduction, we sought to further explore the possible associations between individuals that presented a higher dietary intake of fat, saturated fatty acids, and cholesterol in their diet, with all the anthropometric and analytical data comparing baseline associations and the effect of NPPR-EVOO at the end of the study. Regarding the beginning of the study, a significant and direct association between the serum levels of LDL-c with HbA1c, *p* < 0.05, *r* = 0.375 and with total cholesterol, *p* < 0.001, *r* = 0.870 were observed. Also, there was a direct association between dietary fat with the dietary saturated fatty acids, and dietary total cholesterol, *p* < 0.001, *r* = 0.541. After NPPR-EVOO intake, these strong and direct associations were maintained, except for LDL-c, which was not shown significant, *p* = 0.102.

In an attempt to assess whether the observed differences in lipid profile, HbA1c, and anthropometry were justified because of differences in adherence to MedD, we additionally calculated ROC curves for all the serum analytical and biochemical levels, using adherence to MedD, expressed as PREDIMED score, as a reference to identify individuals with a diet expected to be more balanced and healthier (score ≥ 10), from participants with lower scores (scores < 10); we did not observe any significant correlations with all variables. This means that, in this study, having a MedD does not account for the differences found in the individuals’ serum total cholesterol, LDL-c, HDL-c, and HbA1c. Therefore, having a good adherence to a MedD failed to explain the significant differences the study found in serum lipid profiles and HbA1c after NPPR-EVOO intake, which reinforces the beneficial effect of this Portuguese NPPR-EVOO.

Moreover, the main aim of this study was to explore the biological impact of NPPR-EVOO daily consumption on the clinical, metabolic, and nutritional parameters of healthy volunteers. In line with this aim, a smaller sample of volunteers was grouped with the participants who had not changed their diet (n = 24) throughout the study, nor significantly changed their physical activity (n = 31). This subgroup was further analyzed so that possible confounding effects, besides NPPR-EVOO consumption, would be identified. This subgroup included 23 participants, as two of the participants with unchanged PREDIMED scores significantly changed physical activity (Table 2).

Additionally, we also clustered this subgroup of participants into two clusters according to their PREDIMED score: HH (high-high)—the group of participants that maintained a high adherence to the MedD throughout the study, and LL (low-low)—the group of participants who maintained a low adherence to the MedD during the entire period of the study. In this analysis, it was shown that, regardless of a high or low adherence to the MedD, participants always presented significant changes in HbA1c following NPPR-EVOO; participants in the HH group also showed a significant decrease in LDL-c at the end of the study, and the LL group presented a lower WC after NPPR-EVOO intake. All the other parameters did not show any relevant differences (Table A1 in Appendix A).

The possible potential benefits of NPPR-EVOO in participants that have changed their MedD adherence and physical activity throughout the study was also explored, and although some parameters were significantly changed, it cannot be attributable to NPPR-EVOO consumption (Table A2 in Appendix A). None of the performed analyses found statistical differences involving CRP and NPPR-EVOO.

## 4. Discussion

The MedD, considered a healthy dietary and nutritional pattern, has been associated with a reduced risk of cardiovascular events [18]. Most importantly, health benefits arising from a MedD have been attributed to the high intake of MUFA. Furthermore, the PREDIMED study has shown that EVOO reduces the risk of cardiovascular events compared with participants without EVOO [16]. Additionally, EVOO has been identified as a source of antioxidant, anti-inflammatory, insulin-sensitizing, cardioprotective, antiatherogenic, neuroprotective, immunomodulatory, and anticancer activity. Nonetheless, some studies still lack clarity in unraveling how much of the positive health effects of a MedD are attributable to EVOO intake.

Moreover, some of the clinical studies using EVOO intake are conducted with specific groups of pathologies, such as obesity [32] or individuals at a higher risk of CVD [33,34]. Fewer studies have addressed the effect of EVOO in healthy individuals, without any established disease, and explored its role in analytical parameters and body composition [18,35]; most of them have used intervention periods of 12 weeks to 3 years on healthy, or unhealthy, individuals. A 12-week period was seen to consistently show beneficial effects on HDL-c and LDL-c plasma levels in a dose-dependent manner of the phenolic content in healthy volunteers from the north of Spain [36].

Candido et al., in a randomized controlled study, showed that EVOO reduced body fat mass and improved blood pressure when included in energy-restricted programs for obesity treatment [32]. On the contrary, our study did not find changes in any anthropometric parameters. This could be related to the fact that participants were not obese (BMI ≥ 30 kg/m^2^), as in Candido et al.’s study.

CRP was recently proposed as a stronger predictor of the risk of future cardiovascular events and death than LDL-cholesterol amongst patients receiving statins [37]. This latter association will most probably have implications on treatment beyond statin therapy choices, as a combined use of aggressive lipid-lowering and inflammation-inhibiting therapies might be needed to further reduce atherosclerotic risk and CVD [37]. Our study did not find associations between the serum levels of PCR and LDL-cholesterol. However, individuals assessed in this study did not have a diagnosis of CVD, nor were they taking statins or any other medication. They were recruited from the community and identified as healthy adults, which is probably why their serum levels of inflammation, expressed with CRP, were classified as normal, thus not within a range of levels to be impacted by NPPR-EVOO.

Type-2 diabetes (T2D) is currently estimated to affect 537 million adults worldwide, with a global prevalence of 10.5 percent among adults, and it often appears because of insulin resistance. Diabetes guidelines warrant a broad personalized nutritional approach with recommendations accounting for individual glycometabolic goals. In this context, the MedD has been endorsed as a suitable treatment to help achieve glycemic control, and is of particular interest to the considerable improvement in controlling the glycated hemoglobin (HbA1c) in obese diabetic patients, independent of the weight change [38] with EVOO [39]. Nevertheless, the hypoglycemic effect of EVOO is still not well understood, as some studies do not show any effect [24]. In addition to its role in monitoring glycemia in T2D patients, HbA1c also has been suggested, in population-based studies, to be associated with low-grade inflammation in nondiabetic individuals, associated with higher HbA1c levels and, above all, inflammation, independently of blood glucose concentration [24]. Furthermore, HbA1c has also been pinpointed as a CVD risk factor, most likely due to its inflammation biomarker role [40]. Interestingly, this study has consistently found a significant effect of NPPR-EVOO on lowering HbA1c glycosylation. All participants, regardless of their adherence to the MedD or physical activity status, significantly decreased the glycation of HbA1 upon NPPR-EVOO intake compared with their baseline levels. HbA1c reduction was significant (±0.5%) in the group of individuals that presented irregular dietary behaviors, moving from a good to a low adherence to the MedD, coupled with significant changes in physical activity during the study. These results are in line with others that have observed a decrease in HbA1c (±0.5%) in a group of T2D patients, using a MedD with EVOO, for 24 months [38]. The fact that NPPR-EVOO consumption was associated with a decrease in HbA1C in every participant might probably convey additional health benefits involved with its role in inflammation and oxidative stress.

Violi et al. showed that, after a Mediterranean-type lunch, a significant difference was observed between participants with vs. without EVOO; specifically, LDL-C and its oxidized form were significantly increased in the non-EVOO group. Additionally, when a meal not containing EVOO was given, LDL-C, ox-LDL, and triglycerides increased significantly, whereas HDL-C did not change [18]. Santos et al. showed that individuals with obesity, upon 12 weeks of EVOO intervention, significantly decreased their LDL-c levels to values of approximately 5.11 mg/dL [41]. This is in line with our findings, where LDL-C significantly decreased in volunteers in a magnitude of 9–10 mg/dl upon EVOO intake. Also, in another randomized clinical study, the NUTRAOLEUM study, it was shown that EVOO triterpenes had health benefits (oleanolic and maslinic acid) in addition to their bioavailability and disposition in EVOO [35].

In this study, we have used the PREDIMED score as a tool to assess adherence to a MedD, both at baseline and at the end of the NPPR-EVOO intervention. We observed that, globally, and after the 100 days of NPPR-EVOO intake, participants significantly increased their adherence to thes MedD (score at beginning 9.0 ± 1.7, 3, 12; score at the end 9.7 ± 1.8, 6, 13, *p* = 0.02). NPPR-EVOO intake, as expected, increased the sum of the scores of PREDIMED, most probably because of the extra daily intake of NPPR-EVOO. But, because PREDIMED was not designed as a sensitive tool to detect meaningful changes in dietary habits, this study also evaluated individual dietary intake, assessing the macronutrient profile (total calories, detailed lipid profile, saturated fatty acids, monounsaturated fatty acids, polyunsaturated fatty acids, and total cholesterol) at baseline, and at the end, which could be responsible for the observed metabolic and serum biomarker changes (LDL-cholesterol, HDL-cholesterol, total cholesterol, and glycated HbA1C). No significant changes in the dietary intake of these nutrients were observed, which reinforces the role of EVOO.

Razquin et al. included 4259 individuals, previously from PREDIMED trial, and despite an increase in energy density (due to EVOO intake during the 3-year follow-up trial), whenever the energy increased, a higher adherence to the MedD was seen, and that did not translate into weight gain. This is in line with our results, in which an increase in energy intake did not lead to a higher body fat mass or body weight [42]. Furthermore, MUFAs and PUFAs, important nutrients in the MedD, are known to reduce all-cause-related mortality, as they have been shown to ameliorate glucose metabolism, blood lipids, and CVD risk, observed either in diabetic individuals or in the general population [36]. And so, in this study, the intake in dietary fats, namely, saturated fatty acids, MUFAs, and PUFAs, was analyzed and explored to discover whether they were potentially masking the observed NPPR-EVOO’s beneficial effects. It was seen that, although participants had increased their daily energy intake throughout the study, no significant differences were seen in the profile of fatty acids, MUFAs, PUFAs, and saturated fatty acids. This observation was independent of MedD adherence, level of physical activity, or anthropometry. These results strengthen the idea that the metabolic and analytical changes observed in this study, following NPPR-EVOO intake, cannot be attributable to other dietary lipid modulation, but to NPPR-EVOO intake. It also supports the hypothesis that there is more in EVOO than healthy lipids, as elaborated elsewhere, paving the way for a deeper exploration of EVOO’s bioactive molecules, polyphenol’s role, and EVOO as a functional food item [43].

## 5. Conclusions and Limitations

This study presents some limitations, namely the fact that there were more women than men; also, participants of the study lived in Portugal, a region in which the MedD and EVOO are already common. This might mean that a 100-day intervention might not be long enough to trigger significant changes in body composition when compared to individuals not embedded in a Mediterranean food and gastronomy setting. Further long-term studies, and with non-Mediterranean communities, particularly in patients with CVD and related comorbidities, would add important knowledge regarding the health benefits of EVOO, displaced from the MedD effect. In conclusion, NPPR-EVOO lowered the serum levels of LDL cholesterol and HbA1c, providing clues for the effect of EVOO’s putative health benefits generally, and also on patients with higher cardiovascular and metabolic risk.

## Figures and Tables

**Table 1:** Participants’ clinical and anthropometric characteristics.

Gender (%)	**Men**		**Women**		**Age (Years)**	
	8 (24.2)		25 (75.8)		33.5 ± 11.2 (19–55)	
	**Baseline (before NPPR-EVOO)**		**100 days of NPPR-EVOO**		***p*-Value**	
PREDIMED score						
Low adherence (%)	19 (57.6)		13 (39.4) *		*p* < 0.05	
High adherence (%)	14 (42.4)		20 (60.6) *		*p* < 0.05	
IPAQ score						
Sedentary (%)	3 (9.1)		3 (9.1)		ns	
Irregularly active (%)	5 (15.2)		7 (21.2)		ns	
Active (%)	17 (51.5)		16 (48.5)		ns	
Very active (%)	8 (24.2)		7 (21.2)		ns	
Blood pressure clusters # (mmHg)						
Normal (%)	26 (78.8)		30 (90.9)		ns	
Normal-high (%)	7 (21.2)		3 (9.1) *		*p* < 0.05	
Blood pressure (mmHg)	Diastolic	Systolic	Diastolic	Systolic	Diastolic	Systolic
	116.54 ± 13.22 (96–144)	73.34 ± 8.49 (54–94)	117.54 ± 13.65 (95–141)	73.49 ± 8.34 (60–92)	0.306	0.456
Analytical Data						
HbA1c (%)	5.12 ± 0.32 (4.6–5.8)		4.93 ± 0.24 (4.5–5.6)		0.000 *	
Total cholesterol (mg/dL)	168.22 ± 30.93 (79–200)		162.79 ± 32.30 (84–213)		0.208	
HDL (mg/dL)	62.91 ± 25.70 (34–184)		62.23 ± 24.53 (39–179)		0.281	
LDL (mg/dL)	92.63 ± 24.53 (34–127)		83.23 ± 27.63 (43–138)		0.024 *	
CRP (mg/dL)	0.65 ± 1.37 (0.3–7.7)		0.36 ± 0.20 (0.3–1.17)		0.119	
Dietary intake (n = 32 **)						
Energy (kcal/day)	1394.59 ± 382.30 (422.99–2195.80)		1559.76 ± 459.89 (317.79–2397)		0.004 *	
Lipids (%)	32 ± 7 (18–53)		42 ± 48 (19–305)		0.306	
Saturated fatty acids (g)	9 ± 2 (5–14)		10 ± 3 (5–19)		0.112	
MUFA (%)	11 ± 4 (5–19)		11 ± 4 (3–19)		0.408	
PUFA (%)	5 ± 3 (2–17)		6 ± 3 (2–18)		0.153	
Cholesterol (mg)	26,058.41 ± 18,015.77 (0–72,581.83)		28,473.32 ± 17,401.39 (1130.00–72,275.00)		0.204	
Anthropometry and body composition						
Weight (kg)	65.54 ± 11.94 (48.8–97.5)		65.68 ± 11.45 (49.5–97.1)		0.331	
BMI (kg/m^2^)	23.65 ± 3.19 (18.5–30.0)		23.71 ± 2.97 (18.5–30.0)		0.323	
Circumference (cm)	Female	Male	Female	Male	Female	Male
Waist (cm)	76.69 ± 8.41 (62.5–97.5)	88.96 ± 9.22 (79.4–102)	75.95 ± 7.77 (64.8–92.8)	88.61 ± 8.89 (78.1–102.2)	0.053	0.349
Hip (cm)	94.61 ± 8.17 (83–110.8)	95.88 ± 8.76 (84.5–110)	94.51 ± 6.84 (84–109.6)	97.43 ± 8.23 (87.2–111.8)	0.445	0.010 *
Waist-hip ratio	0.81 ± 0.07 (0.69–0.92)	0.93 ± 0.05 (0.87–1.02)	0.80 ± 0.07 (0.70–1)	0.91 ± 0.07 (0.85–1.02)	0.130	0.030 *
Fat mass (%)	30.08 ± 6.15 (18.10–41.10)	17.50 ± 8.02 (9.80–29.80)	29.91 ± 5.56 (19.90–39.40)	17.96 ± 7.70 (9–29.10)	0.384	0.192
Skeletal muscle mass (kg)	23.10 ± 2.75 (19.50–28.90)	36.88 ± 2.68 (32–39.80)	23.23 ± 2.76 (19.80–29.80)	36.62 ± 2.02 (32.8–39.60)	0.221	0.300
Lean body mass (kg)	39.96 ± 4.29 (34.10–49.10)	61.33 ± 4.18 (53.80–66.30)	40.11 ± 4.34 (19.80–29.80)	60.88 ± 3.24 (54.8–65.60)	0.262	0.244
Dynamometry (kg/force)	21.93 ± 5.86 (12–39)	43.00 ± 6.70 (30–50)	22.04 ± 5.12 (12–34)	43.13 ± 9.34 (30–58)	0.452	0.477

Values are expressed in number of volunteers (n = 33) and percentage or as mean values ± SD (min–max) accordingly; ns—difference was not significant; * *t*-student test—significant *p*-value, *p* < 0.05; # risk groups according to Portuguese Health Board (DGS); ** 24-h recall data referring to a sample of n = 32, as 1 participant had missing values.

**Table 2:** Individuals without changes in their diet (expressed as PREDIMED score) and physical activity throughout the study.

**Analytical Data**	**Baseline (before NPPR-EVOO)**		**100 Days of NPPR-EVOO**		***p*-Value**	
HbA1c (%) (n = 23)	5.10 ± 0.32		4.92 ± 0.24		0.003 *	
Total cholesterol (mg/dL) (n = 22)	177.50 ± 32.88		171.70 ± 37.68		0.300	
HDL (mg/dL) (n = 22)	67.18 ± 29.48		66.73 ± 28.60		0.397	
LDL (mg/dL) (n = 19)	98.91 ± 22.88		87.20 ± 29.93		0.023 *	
CRP (mg/dL) (n = 22)	0.26 ± 0.79		0.14 ± 0.33		0.272	
Dietary intake ** (n = 23)						
Energy (kcal/day)	1440.38 ± 380.172		1605.089 ± 418.120		0.014 *	
Lipids (%)	32.00 ± 7.19		42.13 ± 1.50		0.162	
Saturated fatty acids (%)	3.66 ± 5.60		5.04 ± 5.96		0.217	
MUFA (%)	10.27 ± 4,15		10.18 ± 4.09		0.464	
PUFA (%)	5.73 ± 3.23		5.59 ± 3.61		0.415	
Cholesterol (mg)	28,121.9367 ± 17,745.06031		28,322.9412 ± 17,831.64911		0.480	
Anthropometry and body composition						
Circumference (cm)	Female (n = 17)	Male (n = 6)	Female (n = 17)	Male (n = 6)	Female	Male
Waist (cm)	77.62 ± 7.91	90.08 ± 9.89	76.33 ± 7.97	89.57 ± 10.13	0.011 *	0.279
Hip (cm)	93.62 ± 7.68	95.60 ± 9.52	93.68 ± 6.24	96.97 ± 9.35	0.467	0.004 *
Fat mass (%)	29.04 ± 6.03	17.72 ± 9.45	29.41 ± 5.97	17.58 ± 9.08	0.277	0.322
Dynamometry (kg/force) (n = 23)	21.47 ± 5.06	46.00 ± 3.63	22.82 ± 5.27	46.50 ± 7.99	0.111	0.865

Values are expressed in number of volunteers (n = 23), in percentages or as mean values ± SD (min–max) accordingly; * *t*-student test—significant *p*-value, *p* < 0.05; ** 24-h recall data referring to a sample of n = 23.

## Data Availability

Not applicable.

## Appendix A

**Table A1:** Data characteristics.

Group	Parameter	Baseline	100 Days	*p*-Value
HH (n = 13)	PREDIMED score HbA1c (%)	10.5 ± 0.66 5.08 ± 0.31	11.0 ± 1.1 4.875 ± 0.21	0.069 0.026 *
	Total cholesterol (mg/dL)	189.91 ± 34.51	186.55 ± 33.88	0.263
	HDL (mg/dL)	65.91 ± 15.33	64.64 ± 13.84	0.227
	LDL (mg/dL)	106.38 ± 26.03	93.38 ± 29.20	0.043 *
	CRP (mg/L)	0.03 ± 0.00	0.56 ± 0.9	0.170
	Hand grip strength (kg/strength)	28.00 ± 10.44	28.58 ± 11.58	0.352
	Waist perimeter (cm)	79.78 ± 9.72	78.87 ± 10.45	0.119
	Hip perimeter (cm)	92.26 ± 9.07	92.94 ± 8.55	0.173
	Body fat (%)	24.64 ± 8.71	24.93 ± 8.76	0.320
LL (n = 10)	PREDIMED score HbA1c (%)	7.5 ± 1.7 5.08 ± 0.35	7.5 ± 1.1 4.95 ± 0.28	0.437 0.029 *
	Total cholesterol (mg/dL)	162.20 ± 26.82	159.10 ± 24.77	0.366
	HDL (mg/dL)	69.30 ± 41.88	68.80 ± 41.05	0.441
	LDL (mg/dL)	89.30 ± 20.96	80.70 ± 26.96	0.097
	CRP (mg/L)	0.54 ± 1.14	0.25 ± 0.47	0.257
	Hand grip strength (kg/strength)	28.70 ± 14.30	30.00 ± 13.90	0.215
	Waist perimeter (cm)	83.36 ± 10.13	81.94 ± 10.21	0.006 *
	Hip perimeter (cm)	96.09 ± 6.81	96.09 ± 5.12	0.500
	Body fat (%)	27.30 ± 8.80	27.68 ± 8.85	0.321

* significant *p*-values < 0.05.

**Table A2:** Clinical, dietary, and nutritional characteristics of individuals that changed their diet and physical activity throughout the study.

Group	Parameter	Baseline	100 Days	*p*-Value
LH (n = 4)	HbA1c (%)	5.15 ± 0.22	4.95 ± 0.17	0.117
	Total cholesterol (mg/dL)	192 ± 26.70	181.75 ± 24.34	0.127
	HDL (mg/dL)	63.50 ± 8.31	63.25 ± 8.94	0.380
	LDL (mg/dL)	95.25 ± 23.95	98.50 ± 18.88	0.315
	CRP (mg/l)	0.03 ± 0.0	0.11 ± 0.08	0.195
	Hand grip strength (kg/strength)	22.50 ± 2.87	18.00 ± 2.45	0.001 *
	Waist perimeter (cm)	70.83 ± 1.47	70.58 ± 0.64	0.398
	Hip perimeter (cm)	92.98 ± 3.76	92.95 ± 3.31	0.488
	Body fat (%)	31.00 ± 3.39	32.13 ± 3.00	0.067
	Energy intake (kcal)	1322.55 ± 107.40	1643.39 ± 187.02	0.163
	Lipids intake(kcal)	48.48 ± 4.48	68.02 ± 13.57	0.153
	Saturated fat (kcal)	12.23 ± 1.07	21.49 ± 6.08	0.090
	Cholesterol (mg)	18,867.12 ± 9111.34	29,844.74 ± 10,256.04	0.05 *
HL (n = 3)	HbA1c (%)	5.40 ± 0.06	4.97 ± 0.12	0.040 *
	Total cholesterol (mg/dL)	199.33 ± 5.33	187.00 ± 17.52	0.307
	HDL (mg/dL)	52.67 ± 4.67	55.33 ± 6.98	0.262
	LDL (mg/dL)	120.67 ± 1.76	116.67 ± 1.76	0.352
	CRP (mg/L)	2.59 ± 2.56	0.03 ± 0.00	0.211
	Hand grip strength (kg/strength)	21.33 ± 1.76	23.33 ± 0.67	0.239
	Waist perimeter (cm)	82.43 ± 8.27	80.73 ± 6.82	0.189
	Hip perimeter (cm)	104.93 ± 4.04	100.20 ± 4.95	0.209
	Body fat (%)	36.87 ± 2.95	31.80 ± 1.87	0.108
	Energy intake (kcal)	1275.86 ± 150.65	1424.66 ± 385.90	0.302
	Lipids intake(kcal)	47.72 ± 0.29	52.14 ± 11.73	0.373
	Saturated fat (kcal)	11.95 ± 0.64	15.62 ± 4.88	0.265
	Cholesterol (mg)	25,435.77 ± 9523.71	29,801.11 ± 8304.61	0.233

* significant *p*-values < 0.05.
